# Practical Departments

**Published:** 1905-12-30

**Authors:** 


### PRACTICAL DEPARTMENTS.

### WEBB'S SANITARY LAMPS.

These are apparatus that have been worked out by Mr. J. Webb, the managing director of the Webb Lamp Co., of 11 Poultry, London, E.C., to deal with the problem of the breaking up of noxious gases given off in sewers, lavatories, mortuaries, post-mortem rooms, etc., and they are exceedingly simple in construction. For sewers the Webb lamp forms part of an ordinary street lamp, the illumination being produced by Welsbach incandescent gas mantles, and the lamp itself being covered by a hood which is at the same time a reflector and a thermal insulator for the heat that is produced by the incandescent mantles. The sewer gases are led right into the lamp by means of a pipe forming part of the lamp-post, and they are subjected in the lamp to the action of heat at a temperature of from 500 to 600 degrees F., and also to that of the intense light produced by the burners, before they are allowed to escape into the atmosphere. The result claimed by Mr. Webb is that the whole of the injurious bacteria are destroyed, and the gases pass out into the surrounding atmosphere as hydrogen and oxygen only. For mortuaries, post-mortem rooms, etc., of hospitals, the lamps, as many of them as are necessary, are placed on the ridge of the roof, or in any convenient position, the gases and air from the rooms being led to them, and consumed in the lamps as with the street lamps. We had an opportunity recently of observing the efficiency of Webb's lamps in connection with sewers. A certain watering-place, which shall be nameless, as sewers are never mentioned in health resorts, was distinctly troubled with smells from the street gratings then forming the only means of ventilating the sewers. Some lamps were put up, something on Webb's principle, but not quite. There were the Welsbach burners, the pipe leading the gases up, but unfortunately it led them into the atmosphere below the burners, not into a heated space surrounding them, as Webb does. The smell was less apparent, but it was still there if you knew where to smell for it. Finally some of Webb's lamps were put up, and these were immediately effective, even in the case of a public lavatory open to trippers of all kinds, right on the best part of the shore. The difference can be appreciated now. Where Webb's lamps are there is no smell; where the street gratings are there is much smell often; where the imitation Webb's are there is a smell at times.

### McDOWALL, STEVEN AND CO.'S HEATING AND COOKING APPARATUS.

Messrs. McDowall, Steven and Co., Limited, whose London showrooms are at 4 Upper Thames Street, E.C., and whose works are at Glasgow and Falkirk, where they employ 1,500 hands, make principally heating and cooking apparatus for all purposes. They make a special form of fire-grate, which they call "The Fire on the Hearth Stove," in which there is no draught under the grate, as in the usual form. All the air for combustion passes over the burning fuel. The back of the grate is inclined forwards, and the whole of the back is constructed of fireclay, the space in front inside the fender being fitted with glazed tiles. The tiles and the fireclay become hot, and radiate heat into the room, and the tiles also reflect the heat rays to a large extent. The makers claim also that the arrangement of the grate and the method of burning the fuel helps to consume the smoke that would otherwise be made. The general appearance is very cosy. The firm make the usual pattern of ward stoves in iron and in glazed tiles, with upward or downward flues, in which air from outside is warmed before passing into the ward, while heat is radiated from the fire as well. They make vegetable-steamers having a wheel handle, by means of which all joints are closed at the same time, keeping the apparatus steam-tight; jacketted boilers, with copper covers and balance weights for lifting the covers, the outside of the boiler being lagged with mahogany, much as some steam engines are; every description of cooking-range, for the centre of the kitchen and for the wall, with up or downward flues, and in all of them there are certain features that practical experience has shown to be advantageous; carving tables and hot closets above, the covers of the dishes being fitted with overhead balance gear, enabling any cover to be lifted and lowered quickly, and with a bain marie at one end for keeping certain dishes hot, and many other apparatuses used for heating and cooking.

